# Address of B. H. Catching

**Published:** 1888-11

**Authors:** 


### THE DENTAL REGISTER.

VOL. XLII.] NOVEMBER, 1888. [No. 11.

### COMMUNICATIONS.

### Address of B. H. Catching, President of the Southern Dental Association, Louisville, Ky., Aug. 28, 1888.

[Delivered before the Joint Meeting of the American and Southern Dental Associations.]

IS THE AVERAGE DENTIST OF TO-DAY A SPECIALIST IN MEDICINE?

The question before us is not to determine the relation of dentistry to medicine. That has never been a question with the intelligent dentist, nor with the well informed general practitioner of medicine.

It required not a formal declaration on the part of the medical profession to establish this relation. The very objects and purposes of dentistry as practiced by those competent, denote this. But unfortunately, the number who practice it as specialists of medicine is small, as compared to the number who follow it as a trade.

To the professional class belongs whatever of honor or distinction dentistry has attained.

That the question of our relation to medicine has been asked and even discussed, is not strange, when we consider the great disparity between the number of those who study and practice it as a profession and those who follow it as a trade.

From the latter class has gone forth the impression of the relation existing between profession and trade. In fact, were it not for the comparatively few who demonstrate by practice the relation existing, there could not be found any relation at all.

He who builds a bridge, and welds gold, and feels that with a bottle of creosote or carbolic acid he has reached the highest professional standard, has no conception of the possibilities of his calling.

That is the average dentist of to-day—proficient in that which goes to make the artisan, but deficient in that which makes the specialist in medicine.

Why then should we be surprised that public opinion has caught the idea of our indefinite relation to the medical science. Public opinion is slow to form, but when once formed, it becomes despotic. We have labored under this despotism ever since we created a separate existence, and so long as we continue a separation, so long will our relation be disputed.

I am not here to condemn the non-professional class, but to sympathize with them, for I think a large majority of them are not so from choice, but by the system under which they were educated.

The average man will not rise above his surroundings, nor will he forget his training.

They were not taught in a manner to cause them to look above a trade level, or to appreciate their work as noble and elevating. They were not taught the proud principles and truths of the healing art. They were not taught to feel their equality with other specialists, but rather to feel an ignoble inferiority, even to those whose callings are not more worthy nor more elevating. They were not taught dental surgery in the highest conceptions of the term, but were taught to view the mechanical as the proper basis and highest plane of professional existence.

Such is the limited education of the average dentist of to-day. Don't condemn them, but condemn a system too narrow for the higher purposes of life, a system so contracted as to give only the lowest conceptions of the possibilities of a calling, ranked as one of the three learned professions. If we are to appreciate our opportunities, advantages, and possibilities, we can not continue a plan so deficient in that which relates to professional life.

Our usefulness is not less, but greater than some specialties now listed honorably on the professional calender.

That we have not occupied our proper relation before the world is because of our separate and limited system of education. To condemn a system which has been for a half a century operative, may be thought by some, to say the least, unwise. But wisdom lies in the path of progress and improvement. The advancement now taking place in all relations of life must cause us to keep pace.

For fifty years we have mixed a little of medicine with a little of mechanics, issuing forth, not versed in that which gives us rank and not fully qualified in that which makes our specialty distinctive. Making a dual relation of a single existence has been to our harm.

I do not come to persuade those who are satisfied with the trade idea, for they are past redemption.

I don't come to persuade those who when this questiou is mentioned, cry out " great is Diana, the God of the Ephesians," for they are co-workers with Demetrius.

I do not come to persuade those who have attained to the highest pinnacle of fame without colleges or books for they were born great.

I do not come to persuade those who feel the responsibility of a specialty in medicine resting upon them.

Tell me that dentistry is a specialty in medicine, and yet tell me, as I have heard on the floors of debate, that a medical education is not necessary to practice it as such—that which is the very foundation of the science.

None have risen to distinction possessing only the knowledge necessary for the degree of D.D.S. Many have risen, who, possessing the degree, realize its limited sphere, reached out for that which leads to greater possibilities—a thorough medical education. They stand to-day as leaders in the profession, and upon such must rest the honor of advance in dental science.

I need no better argument for the importance of medical education than to point to the medical degree of our college presidents and professors. He who is my associate on this occasion, a proud dean of a dental college, boasts of no other degree. That which he confers on his graduates he deems not worthy his own use. I applaud him for his wisdom of choice. I feel that in those who have obtained the honorable and only legitimate title in medicine, I must find supporters for my position. It is the only degree known in medicine; the only one that will entitle to rank in that noble calling, the only degree that conveys the full privilege of mdicine and surgery; it is the only degree that can protect the dentist, even in serious results of surgical operations. The law will not hold him blameless who essays to perform operations without this degree, from which evil results follow.

Our peculiar and recently acknowledged relation to medicine must be carefully considered.

The idea of advancement is abroad in the land. I do not suppose a presidential addresss ever struck a more popular chord than did that of the late President of the American Medical Association, the lamented Dr. Garnett. The demand is for higher qualifications, broader curriculum, and an extension of time. The idea of equipping students for so grave and responsible a calling in two short terms, seems to have come suddenly upon the masses as a thing most absurd, so claiming for our specialty an advance in both time and studies, we lay no claims to following suit. For years this has been the uppermost question before us. We have, by the enactment of rigid State laws, shown the conductors of our educational system our disapproval of turning loose hords of graduates without either natural or acquired qualifications.

The very place to guard the sacred precincts of professional life should be at the college doors. Instead we have had to make the line at State borders.

The rapidly multiplying number of schools induces a scramble for matriculates much more befitting a stock exchange than institutions of learning.

I can not but view this increase of schools with alarm. They are not created because of necessity, for necessity does not exist. They are not created for higher aims and purposes, for their acts do not proclaim it. Then why are they created? Sometimes through jealousy, sometimes for personal preferment. I think, more often a better solution is found in the domineering of the trade over the professional spirit.

Tradesmen are not slow to catch the idea of a college here and a college there, believing that the more colleges more students, more trade, which is a legitimate trade principle, but one very detrimental to professional interests. The full solution is found in personal preferment and mercantile interests.

In considering this matter in the light of professional advancement, we are brought face to face with the domineering tendency of the trade concerns of the country, and the patent office relation existing between them. I sometimes compare our situation to that masterpiece in art the "Layocoan." By State laws we throw off the college domination. But there is now upon us this gigantic "trust," which is grinding at the very foundation of professional liberty. Does it not pull down that which we are trying to build up?

The trade idea is encouraged in our ranks, the love of the patent office is fostered, until they have become enemies to true professional dignity.

The average dentist views these as legitimately professional. The medical profession has its Gross, its Sims, its Dalton, but the dental profession has him who made the highest type of operative dentistry possible. Without him, difficulty would stare us in the face and defeat would crown every effort; with him, victory is ours. He left us a legacy that we shall never exhaust, a blessing that shall never end. I allude to Dr. Barnum. Would that his spirit could pervade many of our hearts.

How my very nature did rebel when, before one of the oldest dental societies of the world, a tradesmen read a paper declaring the patent office and the manufacturing establishments to have been the greatest factors in our progress. Without this noble gift of Barnum scarcely one of his patents would be useful. And how my pride was cut when not a single man of that association rose to dispute the assertion.

If they are the truth then what have we been doing in the way of education. I feel that I have not digressed, because these facts relate to our moral status as a profession, and if the average dentist is taught to view them in their true light, they will be the better and dentistry made more honorable.

The relation of dentistry to medicine is that of general surgery. The surgical part of our specialty is its destructive feature. The general surgeon is master of the medical science in so far as it is taught in the medical schools.

The gynæcologist is master of the science of medicine in so far as it is taught in the medical schools.

The ophthalmologist is master of the medical science in so far as it is taught in the medical schools.

And so with the otologist, the dermatologist, the laryngologist. But the dental surgeon is not master of the medical science because he is not taught in the medical schools.

Are you willing to accord more to other specialists than to dental surgeons? Do you rank its importance as less than other specialties? If so, then you may well be content with our separate system of education.

He who cannot see the advantages of an intimate associate relationship of dental surgery to medicine, must be blinded by the darkness of bigotry.

Now that relationship has been defined by the highest medical tribunal in the land, we should set about to qualify for an intimate existence. I do not believe an extension of time under the present system would accomplish what we need or meet the demands placed upon us by an exacting educated public. We must adopt that system which will educate in the science of medicine and perfect us in the art of dentistry.

To do this we must convert the infirmaries of our dental schools into the highest and most comprehensive oral infirmaries, in which the masters should teach from the construction of a rubber denture to the finest bridge work—from the simplest filling to the most elaborate—from the extraction of a tooth to the excision of a maxillary. The qualification for the admission to these infirmaries should be the degree of Doctor of Medicine, conferred by reputable medical colleges.

The transition from the present to the proposed system would be easy. It would necessarily lessen the number of infirmaries; it would materially elevate the standard of education, besides lengthening the time, thereby decreasing the number of matriculates. Besides, a number of the already existing infirmaries would have to close on account of not being able to instruct in the highest type of dental art.

The present system, with the increased number of schools and the rapid advance in the dental art, has made post-graduate schools a necessity. The need for them is now so great that their creation cannot much longer be deferred.

Post-graduate schools would mean the downfall of the present system. The system I propose may be called idealistic. It is sure to be realistic, and then the average dentist will be a specialist in medicine.

